# Co-option of EDM2 to distinct regulatory modules in *Arabidopsis thaliana *development

**DOI:** 10.1186/1471-2229-10-203

**Published:** 2010-09-14

**Authors:** Tokuji Tsuchiya, Thomas Eulgem

**Affiliations:** 1 Center for Plant Cell Biology, Institute for Integrative Genome Biology, Department of Botany and Plant Sciences, University of California at Riverside, Riverside, CA 92521, USA

## Abstract

**Background:**

Strong immunity of plants to pathogenic microorganisms is often mediated by highly specific mechanisms of non-self recognition that are dependent on disease resistance (*R*) genes. The *Arabidopsis thaliana *protein EDM2 is required for immunity mediated by the *R *gene *RPP7*. EDM2 is nuclear localized and contains typical features of transcriptional and epigenetic regulators. In addition, to its role in immunity, EDM2 plays also a role in promoting floral transition. This developmental function of EDM2, but not its role in *RPP7*-mediated disease resistance, seems to involve the protein kinase WNK8, which physically interacts with EDM2 in nuclei.

**Results:**

Here we report that EDM2 affects additional developmental processes which include the formation of leaf pavement cells and leaf expansion as well as the development of morphological features related to vegetative phase change. EDM2 has a promoting effect of each of these processes. While WNK8 seems not to exhibit any vegetative phase change-related function, it has a promoting effect on the development of leaf pavement cells and leaf expansion. Microarray data further support regulatory interactions between WNK8 and EDM2. The fact that the effects of EDM2 and WNK8 on leaf pavement cell formation and leaf expansion are co-directional, while WNK8 counteracts the promoting effect of EDM2 on floral transition, is surprising and suggests that WNK8 can modulate the activity of EDM2.

**Conclusion:**

We propose that EDM2 has been co-opted to distinct regulatory modules controlling a set of different processes in plant immunity and development. WNK8 appears to modulate some functions of EDM2.

## Background

The defense regulator EDM (Enhanced Downy Mildew) 2 was previously shown in Col (Columbia) accessions of *Arabidopsis thaliana *(Arabidopsis) to be specifically required for immunity mediated by the disease resistance (*R*)-gene *RPP (Resistance to Peronospora parasitica) 7 *against the Hiks1 isolate of the pathogenic oomycete *Hyaloperonospora arabidopsidis *(formerly *Peronospora parasitica*; *Hpa*) [1]. Unlike many other plant defense mechanisms *RPP7*-mediated immunity is independent from the defense hormone salicylic acid. Furthermore, *RPP7*-mediated immunity appears thus far to be the only defense mechanism that EDM2 is involved in, as no other EDM2-dependent *R*-gene functions have been reported yet. EDM2 also does not contribute to basal defense, a weaker non-specific plant immune response [2]. Thus, the role of EDM2 in plant defense seems to be restricted to a single or a limited number of defense pathways. The EDM2 protein is nuclear-localized and bears typical features of transcription factors and epigenetic regulators, but does not belong to any established class of plant proteins controlling transcription [1,2]. Consistent with a possible role in transcriptional regulation, EDM2 seems to contribute to disease resistance by promoting transcription of *RPP7 *[1].

We recently reported that a second function of EDM2 is the regulation of flowering time, as *edm2 *mutants exhibit a delay in floral transition [2]. Consistent with this phenotype, transcript levels of the negative flowering regulator gene *FLC *(*Flowering Locus C*) were substantially elevated in *edm2 *mutants. As theses *edm2 *phenotypes are independent from the photoperiod, EDM2 may formally be considered as a member of the autonomous floral promotion pathway, which controls the floral transition by suppressing *FLC *expression independent from day-length [3].

EDM2 interacts in nuclei with the WNK (With No Lysine) 8 protein kinase that can phosphorylate EDM2 [2]. Mutants of this kinase or WNK8 overexpressor lines, however, do not exhibit reduced immunity to *Hpa*Hiks1, but *wnk8 *mutants flower early [2,4]. Double mutant analyses showed that *EDM2 *is epistatic to *WNK8*. Similarly, *FLC *proved to be epistatic to *EDM2*. Thus, WNK8 may act upstream of EDM2 in a regulatory module affecting the floral transition by modulating *FLC *transcript levels [2]. In this model, WNK8 counteracts the promoting effect of EDM2 on flowering.

Here we report that besides its roles in immunity and the floral transition, EDM2 has several additional functions in Arabidopsis development, such as promoting vegetative leaf growth and leaf pavement cell morphology. WNK8 also has a promoting effect on these processes, suggesting that it participates in these EDM2 functions. However, in contrast to its role in the floral transition, WNK8 seems not to counteract the function of EDM2, but to positively contribute to EDM2 activity. Overlapping transcript profiles of *edm2 *and *wnk8 *mutants support common biological roles of both regulators. In addition, EDM2 seems to have a WNK8-independent role in a developmental process related to vegetative phase change. We propose that EDM2 has multiple distinct regulatory functions related to developmental processes and defense. These EDM2 functions differ with respect to their dependency on WNK8. A possible function of WNK8 may be to fine-tune EDM2 activity in some biological processes.

## Results

### *edm2 *and *wnk8 *mutants exhibit a reduction in fresh weight

The transgenic Arabidopsis line *pXVE:HA-EDM2-a *served as a key tool for our previous genetic analysis of *EDM2 *[2]. This line contains in the *edm2-2 *mutant background the estradiol-inducible expression construct *pXVE:HA-EDM2 *encoding the HA epitope tag fused to the N-terminus of the full length EDM2 protein. It exhibits near wild type *EDM2 *and *RPP7 *transcript levels in the absence of estradiol [2]. Furthermore, non-estradiol-treated *pXVE:HA-EDM2-a *plants flower like Col-0 wild type plants and are fully resistant to *Hpa*Hiks1, indicating that un-induced expression of the *pXVE:HA-EDM2 *construct restores wild type *EDM2 *function. Therefore, we used non-estradiol-treated *pXVE:HA-EDM2-a *plants for all complementation experiments described in this study.

**Figure 1:**
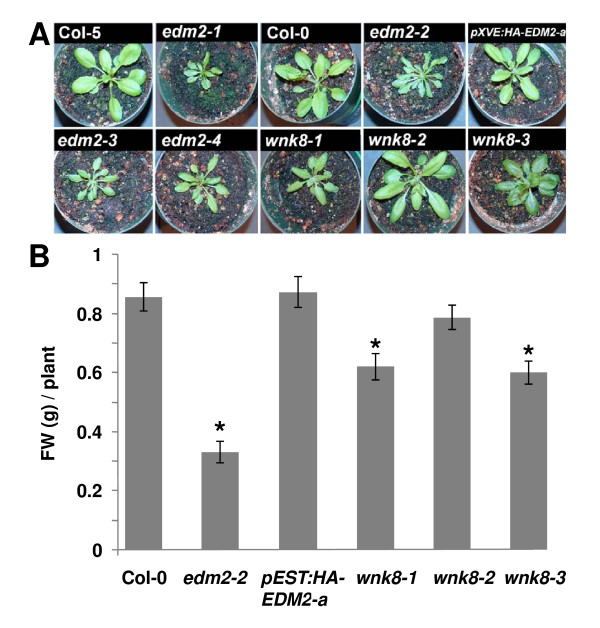
**Mutants of *EDM2 *and *WNK8 *exhibit retarded growth**. **A, B: **Photos of rosettes **(A) **or fresh weight **(B) **of 25 day-old Arabidopsis plants of the indicated genotypes that were simultaneously grown under long-day conditions. Significant deviations (based on *t*-tests; *p *< 0.05) from Col-0 are denoted by asterisks. *pXVE:HA-EDM2*-a plants were not treated with estradiol.

Growing *edm2 *mutants beyond the seedling stage, we observed multiple distinct morphological phenotypes. By the time of bolting, *edm2 *plants appeared smaller due to reduced expansion of the rosette leaves and, consequently, exhibited a significant reduction in fresh weight (Figures 1A &1B). In *pXVE:HA-EDM2-a *plants, wild type growth is restored. Mutants of the EDM2-interacting kinase WNK8 also showed a reduction of leaf expansion and fresh weight. However, this effect was only significant in the *wnk8-1 *and *wnk8-3 *mutants and less pronounced in the *wnk8-2 *mutant, which, compared to *wnk8-1 *and *wnk8-3*, seems to be a weak allele exhibiting a less pronounced reduction of *WNK8 *expression [2].

### *edm2 *and *wnk8 *mutants exhibit altered morphology of leaf pavement cells

**Figure 2:**
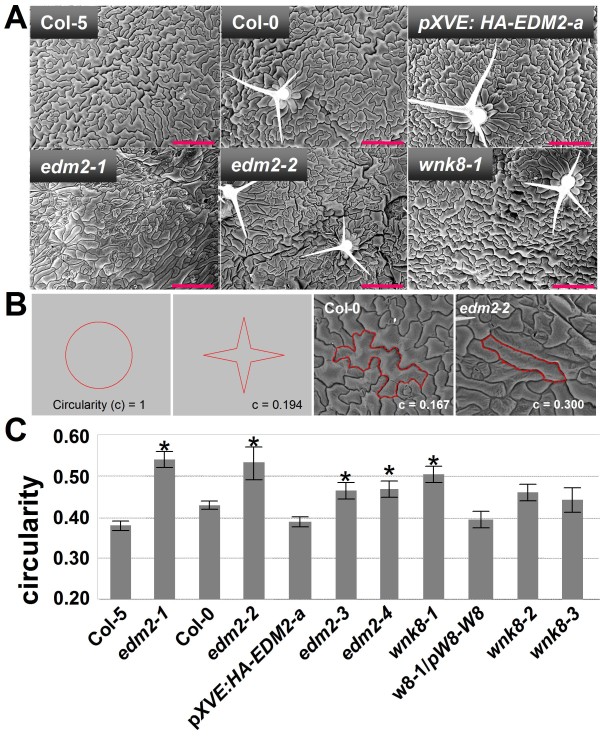
**EDM2 and WNK8 are required for leaf pavement cell development**. **A**: Scanning electron microscopy with adaxial surfaces of the 6th rosette leaves of the indicated Arabidopsis genotypes. 25 day-old plants grown under long-day conditions were used. The red scale bar represents 200 μm. *pXVE:HA-EDM2*-a plants were not treated with estradiol. **B: **Circularity [c = 4pi × (area/perimeter^2^)] as a numerical expression of cell shape. **C: **Average circularity values of 40 - 80 cells per genotype determined from the photos shown in (A) using ImageJ http://rsbweb.nih.gov/ij/. Significant deviations of values from the tested mutants to their respective wild type controls (Col-5 for *edm2-1*, Col-0 for all others) based on *t*-tests (*p* < 0.05) are denoted by asterisks.

We found the morphology of leaf pavement cells to be altered in *edm2 *plants. Typically, the Arabidopsis epidermis consists of interlocked "jigsaw-shaped" cells with protruding lobes and indentations [5,6]. Epidermal cells of *edm2 *mutants are typically of simpler morphology often lacking lobes and indentations (Figure 2A). To quantify this effect we measured the average circularity (c), of all cells within a defined leaf area. Circularity is defined as c = 4pi × (area/perimeter^2^). A perfect circle has a c value of 1.0, while more complex or elongated shapes have lower c values due to decreased area:perimeter ratios (Figure 2B). The pronounced jigsaw shapes of Col-0 epidermis cells are reflected by particularly low c values between 0.1 and 0.2, while the more oval cells often found in *edm2 *epidermis have higher c values. As expected, we found the average c values of each of the four *edm2 *mutants to be significantly higher than those of their respective wild type controls (Col-5 for *edm2-1*; Col-0 for all other lines; Figure 2C). In *pXVE:HA-EDM2-a *plants wild type pavement cell morphology is restored.

Mutations in *WNK8 *weakly affect the morphology of leaf pavement cells. Like those of *edm2 *plants, leaf epidermal cells of *wnk8 *plants tend to be of simpler morphology often lacking lobes and indentations (Figure 2A). Although all three *wnk8 *mutants exhibit elevated circularity values of leaf epidermal cells compared to Col-0, the extent of this effect is weaker than in *edm2 *mutants and only significant in *wnk8-1 *(Figure 2C). Stable transformation of the *wnk8-1 *mutant with a wild type *WNK8 *transgene containing its native promoter (*w8-1*/*pW8-W8*) resulted in low circularity values of leaf pavement cells comparable to those in Col-0.

### Mutation of *EDM2 *causes developmental effects related to vegetative phase change

**Figure 3:**
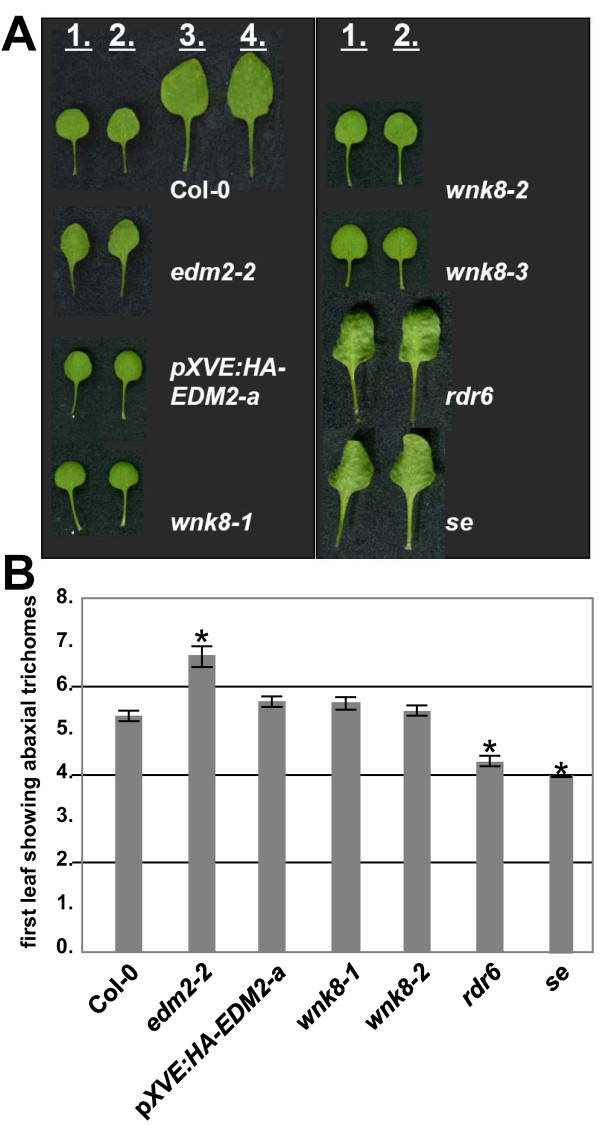
**Vegetative phase change phenotype of *edm2-2 *plants**. **A: **Shown are the first four leaves of 25 day-old Col-0 or the first two leaves of mutant plants grown under long-day conditions. *pXVE:HA-EDM2*-a plants were not treated with estradiol. **B: **First leaf exhibiting abaxial trichomes. Shown are average values based on at least 18 plants with standard errors illustrated as error bars. Plants were grown for 25 days under long day conditions. Significant deviations of values from the tested mutants to Col-0 wild type control plants based on *t*-tests (*p* < 0.05) are denoted by asterisks.

In Col-0 plants, the first two leaves are small and nearly perfectly round with smooth margins. However, *edm2 *plants typically form at this point in their development a pair of small leaves that are "spade-shaped" with slight serrations in the leaf margin (Figure 3A). Their shapes resemble the 2^nd ^pair of leaves found in Col-0, but only the shape and not the size is affected. This morphological phenotype suggested altered vegetative phase change in *edm2 *plants. This developmental process, which is also known as heteroblasty, is defined by changes of leaf anatomy and morphology during the vegetative phase of plant ontogeny [7]. Reminiscent of the known Arabidopsis vegetative phase change mutants *serrate *(*se*), *squint *(*sqn*), *hasty *(*hst*), *zippy *(*zip*), *suppressor of gene silencing 3 (sgs3) *and *RNA-dependent RNA Polymerase 6 (rdr6) *[8-12] the early juvenile phase of vegetative development seemed to be skipped in *edm2 *plants, which is manifested in an apparent omission of the first pair of regular rosette leaves (Figure 3A).

In order to more accurately measure this effect in *edm2-2*, we focused on the production of trichomes as a quantifiable marker of vegetative phase change [13]. Arabidopsis leaves produced during the juvenile phase lack trichomes on their abaxial surface, while the adult phase starts gradually with the production of abaxial trichomes. In Col-0 wild type plants, the onset of abaxial trichome production occurred in leaf 5 or 6, whereas this phase transition was clearly delayed in *edm2-2 *plants (Figure 3B). In contrast, the development of leaves with abaxial trichomes was accelerated in the known phase change mutants *rdr6 *and *se *(Figure 3B). Thus vegetative phase change seems rather to be delayed in *edm2-2 *and EDM2 appears to have a promoting role in this developmental transition. The morphological appearance of early *edm2 *leaves, which suggested the opposite, may not be related to vegetative phase change and may reflect the role of EDM2 in leaf pavement cell development (Figure 2). Both, the morphological phenotype of early *edm2 *leaves and the delayed development of abaxial trichomes are complemented in *pXVE:HA-EDM2-a *plants (Figure 3). None of the three *wnk8 *mutant alleles or three independent WNK8 overexpressor lines, which exhibited clear flowering time phenotypes [2], showed any alterations of the leaf morphology and the vegetative phase transition (Figure 3 and not shown).

In summary, EDM2 is involved in multiple processes that affect distinct aspects of vegetative development, such as vegetative growth, the shape of leaf epidermis cells, and the phase of vegetative leaves. In each case, EDM2 acts as a positive regulator promoting the respective wild type processes. Furthermore, our data suggest that EDM2 and WNK8 act together in mechanisms promoting vegetative growth and epidermal cell development in leaves. As we did not observe any effect of *wnk8 *mutants or *WNK8 *overexpressor lines related to vegetative phase change (this study) or *EDM2/RPP7 *mediated pathogen resistance [2], this protein kinase seems only to contribute to a subset of EDM2 functions.

### *EDM2 *and *WNK8 *have overlapping and co-directional effects on the transcriptome

**Figure 4:**
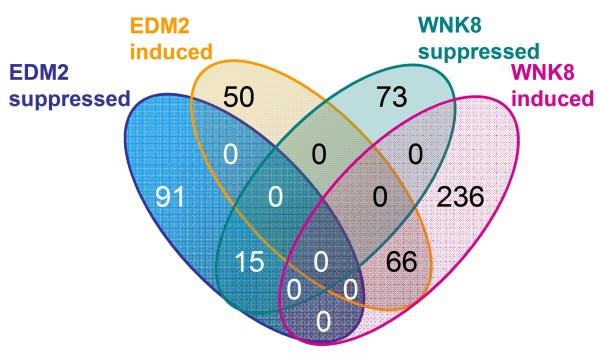
**Venn diagram showing overlaps between the indicated sets of EDM2- or WNK8-dependent expressed genes**.

To assess interactions between *EDM2 *and *WNK8 *at the transcriptome level, we profiled transcript patterns of untreated Col-0, *edm2-2*, *pXVE:HA-EDM2-a*, *wnk8-1*, *wnk8-2*, and *wnk8-3 *seedlings using Affymetrix ATH1 whole genome arrays. For each genotype, signal intensities from three independent biological replicates were averaged and genes identified that were differentially expressed in the *edm2 *or *wnk8 *mutants compared to either Col-0 or *pXVE:HA-EDM2-a *(see methods). Based on these data, we defined six primary sets of EDM2 or WNK8 target genes (Table 1; gene sets 1-6; Additional Files 1, 2, 3, 4, 5, 6) and derived six additional secondary sets of their targets (Table 1; gene sets 7 - 12; Additional Files 7, 8, 9, 10, 11, 12). Gene sets 1 and 2 as well as the secondary gene sets are also represented in Figure 4. The sets "*EDM2 *induced" and "*EDM2 *suppressed" (sets 1 and 2) include genes whose transcript levels in *edm2-2 *compared to Col-0 are significantly lower or higher, respectively. The 222 genes of these two sets represent all genes we found to be differentially affected by EDM2. The more stringently defined sets "core *EDM2 *induced" and "core *EDM2 *suppressed" (Table 1; sets 3 and 4) are limited to those genes whose transcript levels, in addition to being differential in *edm2-2*, are reversed to wild type levels in *pXVE:HA-EDM2-a *plants. As expected, the EDM2 target gene *FLC *[2] is included in the sets "*EDM2 *suppressed" and "core *EDM2 *suppressed". *RPP7 *is not represented on the ATH1 array.

**Table 1:** Sets of genes found to be differentially expressed in *edm2 *or *wnk8 *mutants by microarray experiments

#	gene set name*	Definition**	number of genes***
1	*EDM2*-induced	Col-0 >*edm2-2*	116

2	*EDM2*-suppressed	*edm2-2 *> Col-0	106

3	Core *EDM2*-induced	Col-0 >*edm2-2 ***AND** *pXVE:HA-EDM2-a > edm2-2*	9

4	core *EDM2*-suppressed	*edm2-2 *> Col-0 **AND** *edm2-2 *>*pXVE:HA-EDM2-a*	37

5	*WNK8*-induced	Col-0 >*wnk8-1 ***AND** Col-0 >*wnk8-2 ***AND** Col-0 >*wnk8-3*	302

6	*WNK8*-suppressed	*wnk8-1 *> Col-0 **AND** *wnk8-2 *> Col-0 **AND** *wnk8-3 *> Col-0	88

7	*EDM2 *&*WNK8*-induced	set1 **AND **set 5	66

8	*EDM2 *&*WNK8*-suppressed	set2 **AND **set 6	15

9	only EDM2-induced	set1 **NOT **set5	50

10	only EDM2-suppressed	set2 **NOT **set 6	91

11	only WNK8-induced	set5 **NOT **set1	236

12	only WNK8-suppressed	set6 **NOT **set 2	73

*: refers to the effect of wild type EDM2 or WNK8 on expression of the respective gene set

**: refers to transcript levels of individual genes in the indicated genotypes or presence of genes in defined gene sets: "AND" and "NOT" are used according to Boolean logic.

***: the AGI numbers of these genes and their signal intensities are provided in Additional Files 1, 2, 3, 4, 5, 6, 7, 8, 9, 10, 11 and 12 (Tables S1 - S12).

"*WNK8 *induced" and "*WNK8 *suppressed" (Table 1; sets 5 and 6) represent genes whose transcript levels in each of the three *wnk8 *mutants compared to Col-0 are significantly lower or higher, respectively. The 390 genes of these two sets represent all genes we found to be consistently expressed differentially in all three *wnk8 *mutants. Although this complement of WNK8 target genes (which is based on altered transcript levels in the three mutants) is more stringently defined than the complement of the EDM2 target genes (which is only based on altered transcript levels in one mutant), the former is almost twice as large as the latter, suggesting that WNK8 may have a wider spectrum of biological roles than EDM2. More than a third (36.5%) of the 222 genes differentially regulated by *EDM2 *("*EDM2 *induced" or "*EDM2 *suppressed") are also differentially regulated by *WNK8 *("*WNK8 *induced" or "*WNK8 *suppressed"; Figure 4). Strikingly, all of these 81 genes commonly controlled by *EDM2 *and *WNK8 *are affected by these two regulators in the same manner, as 15 of them are *EDM2 *and *WNK8 *suppressed, while the remaining 66 are *EDM2 *and *WNK8 *induced (Table 1; gene sets 7 & 8). This strict co-directionality of EDM2 and WNK8-dependent transcriptional changes indicates a close functional connection between both regulators and is consistent with the physical interactions between them [2] as well as the fact that they affect a common set of biological processes.

In order to infer possible molecular and/or biological processes controlled commonly by EDM2 and WNK8 or exclusively by each of these two regulators individually we used the "AmiGO" program http://amigo.geneontology.org/cgi-bin/amigo/term_enrichment to identify overrepresented Gene Ontology (GO) terms in gene sets 7 - 12 (defined in Table 1). As shown in Table 2, EDM2 and WNK8 commonly promote expression of genes involved in metabolic processes as well as genes responsive to various stimuli, such as stress, temperature, chemicals and other abiotic cues (Table 2, set 7). Similar themes are found in the sets of genes exclusively and not commonly controlled by EDM2 and WNK8. While the set of "only EDM2-induced" genes is enriched for genes involved in catalytic activity (Table 2, set 9), the set of "only WNK8-induced" genes includes genes responsive to a variety of stimuli, such as stress, temperature, chemicals and abiotic cues (Table 2, set 11). Thus, the roles of EDM2 and WNK8 appear to be partially independent of each other. However, both regulators seem to cooperate regarding a subset of their functions. Interestingly, EDM2 appears to suppress a small set of genes responsive to biotic stimuli and other organisms (Table 2, set 10). This set includes *LURP (Late Upregulated in Response to Hpa)_1 *and *ACD (Accelerated Cell Death) 6*, which we previously found to be *Hpa*-inducible by an EDM2 dependent mechanism [1]. Hence, EDM2 seems to be involved in the *Hpa*-responsive activation of a small set of defense genes as well as their suppression in the absence of *Hpa*.

**Table 2:** Overrepresented GO attributes in sets of differentially expressed genes

Gene set*	GO term (and category)**	p-value	Genes***
set 7 "*EDM2 *&*WNK8*-induced"	metabolic process (BF)	3.08 e-05	INOSITOL(1,4,5)P3 5-PHOSPHATASE II, CKX4, XTH18, CYP86A4, CH1, AT5G42250, AT5G25930, GPAT3, MBF1C, CRK10, AKN2, ATCAD4, JAR1, SHM7, AT5G45650, CYP81F2, WRKY40, ATGA2OX2, AT2G05940, OPR3, AT5G37540, ATHSP101, BMY3, SCL13, GSTU12, APS3, PUB23, AT3G04010, NUDT7, AT2G32150, ATGSTU5, XTR6, AT1G70740, CRK42, GPAT2, RBOHD, AT1G11050, KCS9, ACA1, SZF1

	response to stimulus (BF)	3.01 e-04	INOSITOL(1,4,5)P3 5-PHOSPHATASE II, WRR4, AT4G34150, MBF1C, AT1G59860, JAR1, SHM7, CYP81F2, WRKY40, ATGA2OX2, OPR3, ATHSFA2, ATHSP101, SCL13, CAD1, PUB23, NUDT7, ATGSTU5, TCH4, AT3G09440, RBOHD, KCS9, SZF1

	response to temperature stimulus (BF)	9.73 e-04	AT4G34150, MBF1C, AT1G59860, ATHSFA2, ATHSP101, TCH4, RBOHD, KCS9

	response to stress (BF)	2.10 e-03	WRR4, AT4G34150, MBF1C, AT1G59860, JAR1, CYP81F2, WRKY40, OPR3, ATHSFA2, ATHSP101, PUB23, NUDT7, ATGSTU5, TCH4, RBOHD, KCS9

	response to chemical stimulus (BF)	3.98 e-03	INOSITOL(1,4,5)P3, 5-PHOSPHATASE, II, MBF1C, JAR1, SHM7, WRKY40, OPR3, ATHSFA2, ATHSP101, SCL13, PUB23, NUDT7, ATGSTU5, TCH4, AT3G09440, SZF1

	cellular metabolic process (BF)	5.01 e-03	INOSITOL(1,4,5)P3 5-PHOSPHATASE II, CKX4, XTH18, CYP86A4, CH1, AT5G25930, MBF1C, CRK10, AKN2, ATCAD4, JAR1, SHM7, CYP81F2, WRKY40, ATGA2OX2, AT2G05940, OPR3, ATHSP101, SCL13, GSTU12, APS3, PUB23, NUDT7, ATGSTU5, XTR6, AT1G70740, CRK42, RBOHD, AT1G11050, KCS9, ACA1, SZF1

	response to abiotic stimulus (BF)	7.64 e-03	AT4G34150, MBF1C, AT1G59860, JAR1, ATGA2OX2, ATHSFA2, ATHSP101, PUB23, NUDT7, TCH4, RBOHD, KCS9

	catalytic activity (MF)	3.57 e-09	INOSITOL(1,4,5)P3 5-PHOSPHATASE II, CKX4, XTH18, CYP86A4, CH1, WRR4, AT5G42250, AT4G24160, GPAT3, CRK10, AKN2, ATCAD4, AT1G72520, JAR1, SHM7, AT5G45650, AT5G65140.2, CYP81F2, ATGA2OX2, AT2G05940, OPR3, AT5G37540, ATHSP101, AT1G21120, BMY3, AT5G39580, GSTU12, APS3, PUB23, AT3G04010, SUB, NUDT7, AT2G32150, ATGSTU5, XTR6, TCH4, AT1G70740, AT1G73600, CRK42, GPAT2, RBOHD, KCS9, ACA1

	transferase activity (MF)	7.87 e-05	XTH18, AT4G24160, GPAT3, CRK10, AKN2, JAR1, SHM7, AT2G05940, AT1G21120.1, GSTU12, APS3, SUB, ATGSTU5, XTR6, TCH4, AT1G70740.1, AT1G73600, CRK42, GPAT2, KCS9

set 9 "only *EDM2*-induced"	catalytic activity (MF)	2.60e-06	AT5G39080, AT3G63510, AAE14, PSD2, AT1G22430, SAL1, ATALN, AT5G09300, AT4G31390, AT2G25870, SRS, FC2, AT2G47880, AtCXE17, CSLA11, PKp3, UGT80B1, MCM4, ADC1, AT5G61480, AT2G26870 AT4G33920, CYP78A8, B120, XCP1, ATPREP2, CYP79B2, AT2G04845, AT5G24670, PAL4, CINV2, AT5G38710

set 10 "only *EDM2*-suppressed"	response to biotic stimulus (BF)	1.84 e-03	PCC1, ATCNGC11, ATEBP, FER1, AZI1, PR4, ACD6, pEARLI, 1, PR5, LURP1, MLP34

	response to other organism (BF)	8.71 e-03	PCC1, ATCNGC11, ATEBP, FER1, AZI1, PR4, ACD6, pEARLI, 1, PR5, LURP1

set 11 "only *WNK8*-induced"	Response to stimulus (BF)	6.23 e-15	AT3G42570, GPT2, TIL, STO, CBP60G, BAM1, AT1G51090, AT3G61220, HSP17.6II, OPR2, WRKY18, LOX3, AT2G29500, VTC4, ERD10, AT3G53990, MYB77, CRK11, TCH3, AT1G03220.1, PIL6, CIPK20, WRKY25, WRKY33, BT5, ZAR1, AT3G04210.1, ATHSP90.1, MEK1, ATHB-12, AT5G50915, HSP70, MYBR1, EXO, HSP17.4, LZF1, ATCAMBP25, CMPG1, PHT4;1, AT-HSP17.6A, ATPAP1, ATACA2, SYP122, PAPP2C, LHCB2.3, SOUL-1, TCH2, NHL2, CPK32, CYP707A3, ARR6, SFP1, RAV1, DIN11, AT4G28140, UTR1, LEA14, AT1G55450.1, CEJ1, ATGSTU26, AT1G54050, JAZ6, TIR, NIR1, ADR1, NHL3, AT5G51440, SYP121, TMAC2, CML38, AT5G51190, AT5G54170, ATGSTF8, AT1G19020, ADOF1, CAM9

	response to stress (BF)	1.26 e-13	AT3G42570, TIL, STO, CBP60G, BAM1, AT1G51090, AT3G61220, HSP17.6II, OPR2, WRKY18, LOX3, AT2G29500, VTC4, ERD10, AT3G53990, CRK11, AT1G03220, WRKY25, WRKY33, ZAR1, AT3G04210, ATHSP90.1, MEK1, ATHB-12, HSP70, MYBR1, HSP17.4, ATCAMBP25, AT-HSP17.6A, ATPAP1, SYP122, TCH2, NHL2, CPK32, CYP707A3, DIN11, UTR1, LEA14, AT1G55450, CEJ1, ATGSTU26, AT1G54050, JAZ6, TIR, ADR1, NHL3, AT5G51440, SYP121, TMAC2, CML38, AT5G54170, ATGSTF8, AT1G19020, CAM9

	response to abiotic stimulus (BF)	2.47 e-10	TIL, STO, BAM1, HSP17.6II, AT2G29500, VTC4, ERD10, AT3G53990, TCH3, AT1G03220.1, PIL6, WRKY25, WRKY33, ATHSP90.1, MEK1, ATHB-12, HSP70, MYBR1, HSP17.4, LZF1, ATCAMBP25, AT-HSP17.6A, PAPP2C, LHCB2.3, SOUL-1, TCH2, CPK32, CYP707A3, LEA14, AT1G55450.1, CEJ1, ATGSTU26, AT1G54050, ADR1, AT5G51440, TMAC2, ATGSTF8, CAM9

	response to chemical stimulus (BF)	1.05 e-08	AT3G42570, GPT2, CBP60G, BAM1, WRKY18, AT2G29500, ERD10, MYB77, CRK11, CIPK20, WRKY33, BT5, ATHSP90.1, MEK1, ATHB-12, AT5G50915, HSP70, MYBR1, EXO, ATCAMBP25, CMPG1, AT-HSP17.6A, ATACA2, SYP122, TCH2, CPK32, CYP707A3, ARR6, RAV1, AT4G28140, UTR1, LEA14, CEJ1, ATGSTU26, AT1G54050, JAZ6, NIR1, ADR1, SYP121, TMAC2, AT5G51190, AT1G19020, ADOF1, CAM9

	response to organic substance (BF)	5.50 e-05	GPT2, CBP60G, WRKY18, ERD10, MYB77, CIPK20, WRKY33, BT5, MEK1, ATHB-12, AT5G50915, MYBR1, EXO, CMPG1, SYP122, TCH2, CPK32, ARR6, RAV1, AT4G28140, UTR1, CEJ1, JAZ6, SYP121, TMAC2, AT5G51190, ADOF1, CAM9

	plasma membrane (CC)	4.65 e-03	sks3, GPT2, TIL, AT2G41410, PUP1, AT5G15350, VTC4, TCH3, AtRABA1c, ATHSP90.1, AT1G42470, HSP70, SEC22, AT4G27720, LHCB2.2, ATPAP1, SYP122, PAPP2C, SOUL-1, TCH2, CPK32, SLAH3, AT4G13010.1, AT1G52290.1, TET8, ATPUP18, NHL3, SYP121, CML38, ATGSTF8

*: Gene sets 7 - 12, as defined in table 1 were examined for overrepresented GO attributes using AmiGO. **: GO terms belonging to either of the three GO categories "Biological Function" (BF), "Molecular Function" (MF) or "Cellular Compartment" (CC) are listed. ***: If available common gene names are listed; otherwise AGI gene IDs are given.

## Discussion

This study together with data already published [2] show that the Arabidopsis defense regulator EDM2 has multiple additional roles in development. Its roles are diverse and besides innate immunity, include other apparently unrelated processes, such as development of leaf pavement cells, vegetative phase change as well as control of floral transition.

### EDM2 is involved in several regulatory modules

**Figure 5:**
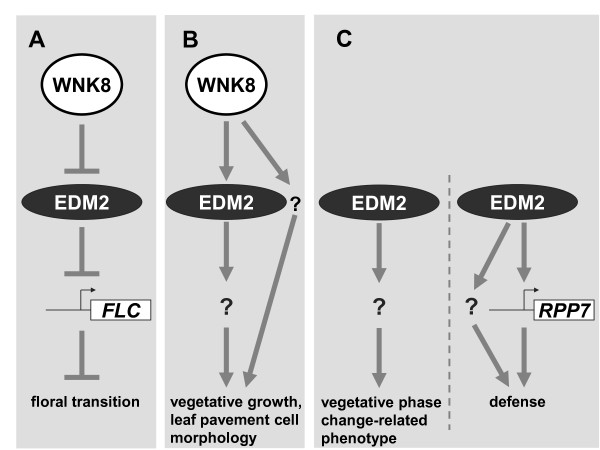
**Different types of EDM2-dependent regulatory modules**. Based on differential effects of EDM2 and WNK8 on developmental or defense processes, three types of EDM2-dependent regulatory modules can be discriminated. **A: **WNK8/EDM2-dependent control of flowering time. Genetic analyses showed that WNK8 operates upstream from EDM2 counteracting the suppressing effect of EDM2 on *FLC *transcript levels. **B: **WNK8/EDM2-dependent control of vegetative growth and epidermal cell morphology. Both WNK8 and EDM2 promote these developmental processes. WNK8 may signal via EDM2 or in parallel to EDM2. **C: **EDM2-dependent control of early vegetative phase change and RPP7- dependent disease resistance to *Hpa*Hiks1. These processes are independent from WNK8. Disease resistance is likely mediated through regulation of *RPP7 *transcript levels. Additional parallel steps may also contribute to this response. **A, B, C: **Arrows indicate promoting effects, while "⊥" symbols indicate suppressing effects.

EDM2 acts together with WNK8 in controlling the floral transition, while WNK8 appears not to have any role in *EDM2*/*RPP7*-mediated disease resistance to *Hpa*Hiks1 [2]. Thus, an EDM2/WNK8-dependent mechanism controls flowering time while a second EDM2-dependent mechanism that seems not to involve WNK8 mediates resistance to *Hpa*Hiks1. Related to the latter regulatory circuit may be the vegetative phase change-related function of EDM2, which is also affected in *edm2 *mutants but not in *wnk8 *mutants or *WNK8 *overexpressor lines. Other roles of EDM2, such as those in vegetative growth and development of leaf epidermal cells may represent a third category of EDM2-dependent regulatory mechanisms, as they seem to involve WNK8. In contrast to the floral transition, however, which is promoted by EDM2 and counteracted by WNK8, vegetative growth and epidermal cell morphology are affected in the same manner by EDM2 and WNK8, which both positively contribute to these developmental processes. Thus, at least three distinct EDM2-dependent regulatory modules that differ in their dependency on and interactions with WNK8 can be discriminated (Figure 5).

### Effects of EDM2 and WNK8 on leaf pavement cell morphology and vegetative growth

Both EDM2 and WNK8 positively contribute to proper development of leaf pavement cells as well as overall vegetative growth. In each case, mutations in *EDM2 *have a stronger impact on the respective phenotype than mutations in *WNK8*. Despite having qualitatively similar effects on leaf pavement cells and vegetative growth, we cannot exclude that EDM2 and WNK8 independently contribute to these developmental processes. Fu et al. [6] found the "jigsaw-like" appearance of Arabidopsis leaf pavement cells to result from the coordinated interplay of the small Rho-type GTPases ROP2 (Rho-related GTPases from plants 2) and ROP4 and their interacting partners RIC1 (ROP-interactive CRIB motif-containing protein 1) and RIC4. In addition, ANGUSTIFOLIA has been found to mediate the formation of protrusions in Arabidopsis leaf cells [14]. However, transcript levels of these developmental regulators appear not to be altered by mutations in *EDM2 *or *WNK8 *in our microarray experiments. Thus, EDM2 and WNK8 may either affect transcript levels of other components of the respective regulatory pathways or affect them by playing roles other than transcriptional regulation.

Besides changes of epidermal cell morphology, mutations in *EDM2 *also impact the overall size and morphology of rosette leaves, which are less expanded in *edm2 *plants compared to Col-0 resulting in a reduced fresh weight of these mutants. This phenotype may be a consequence of the altered leaf pavement cell shape. Several examples indicate causal links between abnormal development of leaf cell morphology and leaf organogenesis [15] and mutations in *ANGUSTIFOLIA *or *ROP2 *also result in more narrow rosette leaves [16,17]. The reduction of leaf size and fresh weight in *wnk8 *mutants is less pronounced than in *edm2 *mutants. Consistent with this, their leaf pavement cell phenotype is also less pronounced compared to *edm2 *mutants.

### Vegetative phase change-related function of EDM2

While *RPP7*-mediated resistance to *Hpa*Hiks1 is strongly dependent on EDM2 [1], this immune response appears not to require WNK8. Similarly, the vegetative phase change-related phenotype of *edm2 *mutants appears to be absent in any of the *wnk8 *mutants or *WNK8 *overexpressor lines. Thus, the roles of EDM2 in *RPP7*-mediated resistance and vegetative phase change appear to be distinct from its roles in the control of flowering time, leaf pavement cell development and vegetative growth. A pathway for the biogenesis of *trans-acting *siRNAs involving *HASTY*, *ZIPPY*, *SGS3 *and *RDR6 *has been implicated in the control of early vegetative phase change in Arabidopsis. However, neither transcript levels of these genes nor of five genes that seem to be controlled by this pathway (*ARF3 *[*AUXIN RESPONSE FACTOR 3*; At2g33860], *ARF4 *[*AUXIN RESPONSE FACTOR 4*, At5g60450], *SPL3 *[*SQUAMOSA PROMOTER BINDING PROTEIN-LIKE 3*, At2g33810], At1g63130 and At5g18040) [12] appear to be affected by EDM2 in our microarray experiments. Thus, involvement of EDM2 in this siRNA pathway seems unlikely.

### EDM2/WNK8-dependent regulation of floral transition

We previously found EDM2 and WNK8 to operate in a regulatory module controlling flowering time [2]. EDM2 has a promoting effect on this developmental transition, while WNK8 appears to counteract this EDM2 function. Given their antagonistic effects on the floral transition, the co-directionality of EDM2 and WNK8-dependent transcriptome changes seems counter-intuitive. However, our microarray analyses were performed with vegetative tissues prior to the floral induction. Thus, transcriptional effects caused by EDM2- or WNK8-modulated *FLC *activity should not be represented in our microarray data set.

As we describe previously [2], the role of EDM2 in promoting the floral transition by suppressing the floral repressor *FLC *in a photoperiod-independent manner resembles that of the well-characterized autonomous floral promotion pathway [3]. So far seven regulatory genes have been associated with this pathway, *FCA *(At4g16280), *FY *(At5g13480), *FLK (FLOWERING LOCUS KH DOMAIN*, At3g04610), *FPA *(At2g43410), *FLD (FLOWERING LOCUS D*, At3g10390), *FVE *(At2g19520) and *LD (LUMINIDEPENDENS*, AT4G02560) [18-21]. We did not observe in our microarray experiments differential effects on the transcript levels of any autonomous pathway gene in *edm2-2 *ruling out that *EDM2 *affects this pathway simply by controlling the expression of one of its known components. A role of EDM2 as a member of the autonomous pathway is further supported by our microarray data, which revealed substantial overlap between transcriptional signatures of the *fpa/fld *double mutant [3] as well as the *edm2 *and *wnk8 *mutants (Additional File 13, Figure S1). The fact that autonomous pathway mutants (like *edm2 *mutants) exhibit delayed vegetative phase change is also consistent with the proposed function of EDM2 as a new component of this floral promotion pathway [13].

### Co-option of EDM2 to distinct regulatory processes

According to whole genome sequences available from Genbank http://blast.ncbi.nlm.nih.gov/Blast.cgi, EDM2-like proteins (ELPs, as defined by [1]) appear to have originally evolved from a common ancestor of land plants, as they are present in eudicots, monocots and bryophytes, but are lacking in prokayotes, green algae, fungi, protozoans or metazoans. While key mechanisms of plant development must have emerged early in the lineage of land plants, the acquisition of specific mechanisms of pathogen recognition and defense activation are likely more recent events resulting from co-evolution of higher plants along with pathogens adapting to their hosts [22]. Thus, we believe that at least some of the developmental roles of EDM2 reflect more ancient functions than its role in defense. Thus, EDM2 as a regulator of some developmental processes may have been co-opted to control additional functions including defense. Co-option, the recruitment of extant genes to new functions [23], often involves the utilization of new promoter elements leading to a wider spectrum of expression and, consequently, deployment of the respective gene products into new regulatory or metabolic contexts. Alternatively, new domains can be added to an extant protein enabling it to execute new molecular roles in addition to its ancestral functions. Of interest in this respect may be the C-terminal extension of EDM2, which we found to act as a potent transcriptional activation domain (T.T. & T.E. unpublished). This C-terminal extension is only present in ELP family members of the eudicot clade [1]. Thus, the relatively recent acquisition of the C-terminal extension may be responsible for a widened functional spectrum of EDM2. Future studies will have to address if EDM2 orthologs that lack this domain have a reduced set of EDM2-related roles.

At least some EDM2 functions must be occurring simultaneously in the same cell types. EDM2 and WNK8 transcripts are detectable in all aerial vegetative tissues as well as in the shoot apex during and after the floral transition (AtGenExpress; http://jsp.weigelworld.org/expviz/expviz.jsp). As most vegetative aerial Arabidopsis tissues appear to be capable of defense responses to *Hpa *[24], roles of EDM2 in immunity are likely to spatially coincide with its roles in vegetative phase change or leaf pavement cell development and vegetative growth. Co-option of EDM2 to multiple simultaneous functions may reflect an important adaptation allowing for crosstalk between apparently unrelated biological processes. For example, limited amounts of cellular EDM2 protein may constitute a "regulatory bottleneck" enabling the plant to balance the allocation of its resources between defense responses and different developmental processes.

Additionally, as discussed elsewhere [2], EDM2-dependent developmental processes may be manipulated by biotrophic pathogens, such as *Hpa*Hiks1. A typical feature of pathogens that are well adapted to their hosts is the use of effector molecules, which they secrete into host cells to increase pathogen virulence. Such effectors or virulence factors can attenuate host defense processes or change plant metabolism or development [25] in order to generate a more favorable environment for the pathogen in the host. Often plants have evolved mechanisms to recognize effector-mediated interferences with their regulatory apparatus to activate strong immune responses [26,27]. A similar scenario may explain the involvement of EDM2 in developmental and immune processes.

## Conclusions

Besides *RPP7*-mediated immunity, EDM2 positively affects several developmental processes which include the formation of leaf pavement cells, leaf expansion, a role related to vegetative phase change as well as floral transition. These EDM2-dependent processes differ with respect to their dependency on the EDM2-interating protein kinase WNK8. While WNK8 seems not to affect the vegetative phase change-related role of EDM2, it has a promoting effect on the development of leaf pavement cells and leaf expansion. The fact that the effects of EDM2 and WNK8 on leaf pavement cell formation and leaf expansion are co-directional, while WNK8 counteracts the promoting effect of EDM2 on the floral transition suggests that WNK8 can modulate the activity of EDM2. Thus, we propose that EDM2 has been co-opted to distinct regulatory modules controlling a set of different processes in plant immunity and development. Some functions of these EDM2 functions appear to be modulated by WNK8.

## Methods

### Mutant lines

Wild-type plants are the *Arabidopsis *accession Col-0 and Col-5 which were obtained from the Arabidopsis Biological Resource Center (ABRC). The *edm2-1*, *edm2-2*, *edm2-3 *and *edm2-4, pXVE:HA-EDM2-a, wnk8-1, wnk8-2, wnk8-3, rdr6 *(*sgs2-1*) and *se *mutant lines were described previously [1,2,4,28,29]. Briefly: The *edm2-1 *mutant contains a fast neutron-induced deletion of 2 bp creating a premature stop codon predicted to result in a truncated protein product. The *edm2-2*, *edm2-3 *and *edm2-4 *SALK T-DNA lines contain insertions in the 4^th ^and 5^th ^of 17 introns (*edm2-2 *&*edm2-3*, respectively) or the 9^th ^of 18 exons (*edm2-4*). While *edm2-2 *appears to be an mRNA null allele, low transcript levels of *edm2-1 *are detectable. Transcript levels of *edm2-3 *and *edm2-4 *are partially reduced (T.T. & T.E. unpublished). The *wnk8-1 *and *wnk8-2 *SALK T-DNA mutants contain insertions in the 5^th ^and 7^th ^of seven exons, respectively, resulting in reduced levels of *WNK8 *transcript. The *wnk8-3 *GABI-Kat T-DNA mutant harbors an insertion in the 2^nd ^of six introns resulting in strongly reduced *WNK8 *transcript levels. The *se *mutant was generated by X-ray mutagenesis of Col-1 plants and contains a 7 bp deletion causing a frameshift altering the last 27 amino acids of the predicted Serrate protein.

### Transgenic lines

For the functional complementation of the *wnk8-1 *mutation, genomic DNA fragments containing the *WNK8 *gene as well as its entire upstream intergenic region were PCR-amplified using primers, gWNK8GW-F (5'-GGGGACAAGTTTGTACAAAAAAGCAGGCTGCTCTTACGTACATCGTCTTTTTCAC-3') and gWNK8GW-R (5'- GGGGACCACTTTGTACAAGAAAGCTGGGTATCAAGAGATGTTAACTGCTTTTTGC-3'). The amplified genomic DNA fragment was introduced into pDONR/Zeo plasmid (Invitrogen) to generate a Gateway entry clone and the cloned *WNK8 *genomic fragment was transferred into the pEarleyGate302 vector [30]. the resulting construct was transformed into *wnk8-1*.

### Microscopy

In order to observe the shape of leaf epidermal pavament cells, scanning electron microscopy was performed with fresh 6^th ^rosetta leaves of wild type or mutant lines using a Hitachi TM-1000 scanning electron microscope (Hitachi High-Technologies). Individual images were processed using Image J software http://rsb.info.nih.gov/ij/ to measure circularity. The appearance of abaxial trichomes was monitored using a stereomicroscope.

### Microarray analysis

Arabidopsis plants were grown on soil under short day conditions (8 h light/16 h dark, 22°C and light intensity of 120 umol m^-2^s^-1^). 2-week-old seedlings were harvested 7 to 8 hour after dawn and total RNA was isolated using TRIzol Reagenet (Invitrogen). RNA was further purified with RNeasy Plant mini kit columns (Qiagen) according to the protocols supplied by the manufacturers. The purified RNA was processed and hybridized to the Affymetrix Arabidopsis ATH1 genome array GeneChip following the manufacturer's instructions (Affymetrix) by the IIGB Core Instrumentation Facility of University of California at Riverside. Three independent biological replicates were performed for each treatment. Data was processed in the statistical programming environment R using Bioconductor packages. Raw expression values were normalized using the robust multichip averaging algorithm. Analysis of differentially expressed genes (DEGs) was performed with the LIMMA package [31]. The Benjamini and Hochberg method was selected to adjust *P *values for multiple testing [32]. Differentially expressed genes were ranked using the B statistics, and these exhibiting a B-statistic of greater than 0 were considered differentially expressed. Venn diagrams were constructed using the Venny tool http://bioinfogp.cnb.csic.es/tools/venny/index.html. The AmiGO program http://amigo.geneontology.org/cgi-bin/amigo/term_enrichment was applied to identify enriched Gene Ontology (GO) terms in sets of co-expressed genes. The following AmiGO program settings were used: "Database Filter": "TAIR"; "Maximum p-value": "0.01"; Minimum number of gene products": "10% of input gene set".

### Accession Numbers

Sequence data from this article can be found in the Arabidopsis Genome Initiative or GenBank/EMBL databases under the following accession numbers: *EDM2 *(AT5G55390), *WNK8 *(AT5G41990). The microarray data have been deposited in MIAME-compliant format in the GEO database under the accession number GSE17499.

## Authors' contributions

TT designed and performed all experiments and wrote parts of the manuscript. TE supervised this project and wrote parts of the manuscript. All authors read and approved the final manuscript.

## Supplementary Material

Additional file 1 **Table S1: Microarray Data Set 1**. List of genes in microarray data set 1 (as defined in Table 1). Click here for file

Additional file 2 **Table S2: Microarray Data Set 2**. List of genes in microarray data set 2 (as defined in Table 1). Click here for file

Additional file 3 **Table S3: Microarray Data Set 3**. List of genes in microarray data set 3 (as defined in Table 1). Click here for file

Additional file 4 **Table S4: Microarray Data Set 4**. List of genes in microarray data set 4 (as defined in Table 1). Click here for file

Additional file 5 **Table S5: Microarray Data Set 5**. List of genes in microarray data set 5 (as defined in Table 1). Click here for file

Additional file 6 **Table S6: Microarray Data Set 6**. List of genes in microarray data set 6 (as defined in Table 1). Click here for file

Additional file 7 **Table S7: Microarray Data Set 7**. List of genes in microarray data set 7 (as defined in Table 1). Click here for file

Additional file 8 **Table S8: Microarray Data Set 8**. List of genes in microarray data set 8 (as defined in Table 1). Click here for file

Additional file 9 **Table S9: Microarray Data Set 9**. List of genes in microarray data set 9 (as defined in Table 1). Click here for file

Additional file 10 **Table S10: Microarray Data Set 10**. List of genes in microarray data set 10 (as defined in Table 1). Click here for file

Additional file 11 **Table S11: Microarray Data Set 11**. List of genes in microarray data set 11 (as defined in Table 1). Click here for file

Additional file 12 **Table S12: Microarray Data Set 12**. List of genes in microarray data set 12 (as defined in Table 1). Click here for file

Additional file 13 **Figure S1: Venn diagrams**. Venn diagrams representing overlapping sets of *EDM2/WNK8 *controlled genes and genes controlled by the autonomous pathway genes *fpa*, *fd *and *fld*. Click here for file
