# Supplementary figures and images for: Co-option of EDM2 to distinct regulatory modules in Arabidopsis thaliana development
Source: BMC Plant Biol. 2010 Sep 14;10:203. doi: 10.1186/1471-2229-10-203 (PMC2956552; doi:10.1186/1471-2229-10-203)

## Slide 1
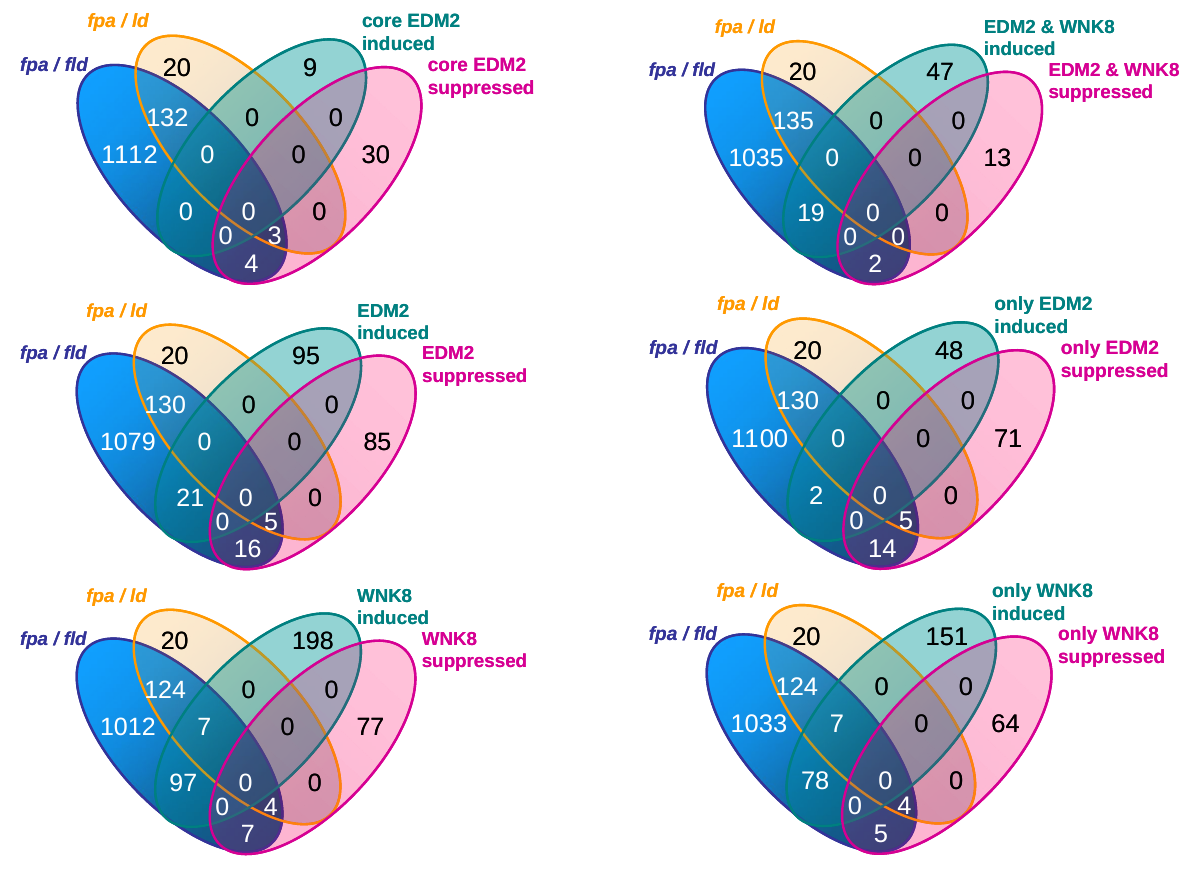

Supplement: Additional file 13 — Figure S1: Venn diagrams. Venn diagrams representing overlapping sets of EDM2/WNK8 controlled genes and genes controlled by the autonomous pathway genes fpa, fd and fld. [file 1471-2229-10-203-S13.PPT]
